# 

**Published:** 1834-01-01

**Authors:** 


### CONTENTS

OF THE

### MEDICO-CHIRURGICAL REVIEW,

### No. XXXIX. JANUARY 1, 1834.

### REVIEWS.

I.

MEDICO-CHIRURGICAL TRANSACTIONS, (Vol. XVIII. Part I.)

I. Cases and Observations connected with Disease of the Pancreas. By Dr. BRIGHT .. .. .. .. .. .. .. .. .. .. .. .. .. .. .. .. 1

II. Additional Facts respecting Glanders in the Human Subject. By Dr. ELLIOTSON .. .. .. .. .. .. .. .. .. .. .. .. .. .. .. .. 10

III. Cases of Sloughing Abscess connected with the Liver, &c. By CÆSAR HAWKINS, Esq. .. .. .. .. .. .. .. .. .. .. .. .. .. .. 11

IV. On the Ulcerative Process in Joints. By C. ASTON KEY, Esq.. .. .. .. .. 25

II.

The History of a Case in which Animals were found in Blood drawn from the Veins. By J. S. BUSHNAM, Esq.. .. .. .. .. .. .. .. .. .. .. .. .. 38

III.

A Treatise on Diseases of the Eye. By W. LAWRENCE, F.R.S.. .. .. .. .. .. 40

IV.

A Dictionary of Practical Medicine, &c. By JAMES COPLAND, M.D. .. .. .. 46

V.

A Treatise on those Diseases of the Brain and Nervous System, usually called Mental. By D. UWINS, M.D. .. .. .. .. .. .. .. .. .. .. .. .. .. .. 49

VI.

A compendious History of Small-pox, with an Account of a Mode of Local Treatment which prevents Scarring. By H. GEORGE, Surgeon .. .. .. .. .. 59

VII.

Illustrations of the Effects of Poisons. By G. LEITH ROUPELL, M.D. .. .. .. 62

VIII.

WORKS ON MORBID ANATOMY.

I. Principles and Illustrations of Morbid Anatomy, &c. By Dr. HOPE .. .. }

II. Illustrations of the Elementary Forms of Disease. By Dr. CARSWELL.. .. } 66

III. Anatomie Pathologique. By J. CRUVEILHIER, M.D. .. .. .. .. .. .. }

1. The Mucous Glands in Malignant Cholera .. .. .. .. .. .. .. .. .. 67

2. Physical Characters of Carcinoma .. .. .. .. .. .. .. .. .. .. .. 68

3. Physiological Character of Ditto .. .. .. .. .. .. .. .. .. .. .. 73

4. Cruveilhier on the Pathology of Cholera.. .. .. .. .. .. .. .. .. .. 75

IX.

A Memoir on the Advantages and Practicability of dividing the Stricture in Strangulated Hernia. By C. ASTON KEY, Esq. .. .. .. .. .. .. .. .. .. 80

X.

1. Observations on Obstetric Auscultation. By E. KENNEDY, M.D. .. .. .. .. 92

2. Signs of Pregnancy and Delivery. By W. F. MONTGOMERY, M.D. .. .. .. 124

Tangible Signs of Pregnancy.. .. .. .. .. .. .. .. .. .. .. .. .. .. 98

Audible Signs of Pregnancy .. .. .. .. .. .. .. .. .. .. .. .. .. .. 100

Compound Pregnancy .. .. .. .. .. .. .. .. .. .. .. .. .. .. .. 112

Complicated Pregnancy .. .. .. .. .. .. .. .. .. .. .. .. .. .. .. 113

Pseudo-Pregnancy .. .. .. .. .. .. .. .. .. .. .. .. .. .. .. .. 114

Simulated Pregnancy .. .. .. .. .. .. .. .. .. .. .. .. .. .. .. 119

Moles or False Conception .. .. .. .. .. .. .. .. .. .. .. .. .. .. 124

On the Corpora Lutea .. .. .. .. .. .. .. .. .. .. .. .. .. .. .. 127

XI.

A rational Exposition of the Physical Signs of Diseases of the Lungs, &c. By C. D. Williams, M.D. .. .. .. .. .. .. .. .. .. .. .. .. .. .. 132
On the Motions and Sounds of the Heart .. .. .. .. .. .. .. .. .. .. .. 133

XII.

Surgical Essays, the Result of Clinical Observations made at Guy's Hospital. By B. B. Cooper, F.R.S. &c. &c. .. .. .. .. .. .. .. .. .. .. .. 137

### PERISCOPE.

### PART I.

### Spirit of the English Periodicals, and Notices of English Medical Literature.

1. The Treatment of Asiatic Cholera and Chronic Diarrhœa. By Mr. Langford.. 145

2. On Nervous Affections of Voluntary Muscles. By Mr. Ed. Lee.. .. .. .. 148

3. A Compendium of Osteology. By Dr. Witt. .. .. .. .. .. .. .. .. 149

4. The Preceptor, Didactic and Clinical, No. I. .. .. .. .. .. .. .. .. 151

The Introductories .. .. .. .. .. .. .. .. .. .. .. .. .. .. .. .. 152

A. Dr. Grant .. .. .. .. .. .. .. .. .. .. .. .. .. .. .. .. .. 152

B. Dr. Watson .. .. .. .. .. .. .. .. .. .. .. .. .. .. .. .. .. 153

C. Mr. Wardrop .. .. .. .. .. .. .. .. .. .. .. .. .. .. .. .. 153

D. Mr. Guthrie .. .. .. .. .. .. .. .. .. .. .. .. .. .. .. .. 153

5. Mr. Robertson on Cynanche Laryngea .. .. .. .. .. .. .. .. .. .. 155

6. Dr. Maxwell on Uterine Hæmorrhage .. .. .. .. .. .. .. .. .. .. 156

7. Mr. Cunningham on Retroversio Uteri .. .. .. .. .. .. .. .. .. .. 156

8. Mr. Easton on Diseases of the Poor .. .. .. .. .. .. .. .. .. .. 158

9. Dr. Davis on Sterility .. .. .. .. .. .. .. .. .. .. .. .. .. .. 159

10. Discharge of a Fœtus from the Umbilicus .. .. .. .. .. .. .. .. .. 163

11. Crying and Breathing of the Fœtus in Utero.. .. .. .. .. .. .. .. .. 163

12. Difference between the fœtal and perfect Heart .. .. .. .. .. .. .. .. 163

13. Dr. Kennedy on Umbilical Hernia of the Gravid Uterus .. .. .. .. .. .. 164

14. Auscultation in Still-born Children .. .. .. .. .. .. .. .. .. .. .. 164

15. Dr. Fletcher's Case of Apoplectiform Disease .. .. .. .. .. .. .. .. 164

16. Wound of the Gluteal Artery. By Mr. Carmichael .. .. .. .. .. .. .. 165

17. On fungating Venereal Ulcer .. .. .. .. .. .. .. .. .. .. .. .. .. 167

18. Bony Union of Fracture of the Cervix Femoris .. .. .. .. .. .. .. .. 170

19. Dr. Patterson on Mammary Irritation .. .. .. .. .. .. .. .. .. .. .. 170

20. Case of Œsophagotomy .. .. .. .. .. .. .. .. .. .. .. .. .. .. 172

21. Deep-seated Nævi treated by Seton .. .. .. .. .. .. .. .. .. .. .. 174

22. Case of large Ovarian Tumour.. .. .. .. .. .. .. .. .. .. .. .. .. 177

23. A. Ascites apparently cured after Twelve Tappings .. .. .. .. .. .. .. 177

B. Natural Paracentesis Thoracis .. .. .. .. .. .. .. .. .. .. .. .. 178

C. Extraordinary Case of Pericarditis.—Dr. Dickson .. .. .. .. .. .. .. 178

24. Philadelphia Report on Cholera .. .. .. .. .. .. .. .. .. .. .. .. 178

25. Ricean Etiology of Cholera .. .. .. .. .. .. .. .. .. .. .. .. .. 180

The Bengal Proteus!.. .. .. .. .. .. .. .. .. .. .. .. .. .. .. .. 181

26. Bristol Medical School .. .. .. .. .. .. .. .. .. .. .. .. .. .. 181

Dr. Carrick's important Remarks on Medical Education .. .. .. .. .. .. 182

27. Remarkable Case of Wounded Intestine. By Mr. Davids.. .. .. .. .. .. 182

28. The Stomach-pump in Vesical Hæmorrhage. By Mr. Simpton .. .. .. .. 183

Medical Politics.

29. The Aldersgate Question .. .. .. .. .. .. .. .. .. .. .. .. .. .. 185
Resolutions of the Westminster Medical Society.. .. .. .. .. .. .. .. .. 185
Remarks on the Transaction in general.. .. .. .. .. .. .. .. .. .. .. 186
Medical Nepotism .. .. .. .. .. .. .. .. .. .. .. .. .. .. .. .. 187
30. Liverpool Medical Society.. .. .. .. .. .. .. .. .. .. .. .. .. .. 187
Dr. Baird's Case of Spontaneous Gangrene .. .. .. .. .. .. .. .. .. 187
31. Bristol Medical Conversation Society—Dr. Davies' Cases .. .. .. .. .. .. 188
32. Naval Medical Officers .. .. .. .. .. .. .. .. .. .. .. .. .. .. 188
33. The late Joshua Brookes .. .. .. .. .. .. .. .. .. .. .. .. .. .. 189

34. Obituary—extraordinary Death of our oldest Contemporary .. .. .. .. .. 190
35. Amputation in an Infant .. .. .. .. .. .. .. .. .. .. .. .. .. .. .. 191
36. Mysterious Case of Pulmonary Abscess .. .. .. .. .. .. .. .. .. .. .. .. 192

### Spirit of the Foreign Periodicals.

1. Acetas Plumbi in Pulmonary Affections.. .. .. .. .. .. .. .. .. .. .. 193

2. Re-vaccination in the Prussian Armies .. .. .. .. .. .. .. .. .. .. .. .. 195

3. Sudden Death during Intoxication .. .. .. .. .. .. .. .. .. .. .. .. .. 196

4. On the Medicinal operation of Whey in Chronic Diseases, especially Phthisis 197

5. Enormous enlargement of the Uterus .. .. .. .. .. .. .. .. .. .. .. .. 200

6. Abnormal Flexure of the Colon .. .. .. .. .. .. .. .. .. .. .. .. .. 200

7. Curious Otitis in the Insane .. .. .. .. .. .. .. .. .. .. .. .. .. 201

8. The Vapour Cave at Pyrmont .. .. .. .. .. .. .. .. .. .. .. .. .. 203

9. New Lithotome .. .. .. .. .. .. .. .. .. .. .. .. .. .. .. .. .. 203

10. Continuance of Life after Destruction of Brain .. .. .. .. .. .. .. .. 204

11. Thickness of Sac in Femoral Hernia .. .. .. .. .. .. .. .. .. .. .. 204

12. Venereal Condylomata .. .. .. .. .. .. .. .. .. .. .. .. .. .. .. 204

13. Facial Hemiplegia .. .. .. .. .. .. .. .. .. .. .. .. .. .. .. .. 204

14. Iodine against Salivation .. .. .. .. .. .. .. .. .. .. .. .. .. .. 205

15. Poisoning by Hyosciamus .. .. .. .. .. .. .. .. .. .. .. .. .. .. 205

16. Encysted Dropsy, spontaneous cure .. .. .. .. .. .. .. .. .. .. .. 206

17. Ligature of the Subclavian Artery .. .. .. .. .. .. .. .. .. .. .. .. 206

18. Cæsarian operation .. .. .. .. .. .. .. .. .. .. .. .. .. .. .. .. 207

19. The artificial production of Vaccine .. .. .. .. .. .. .. .. .. .. .. 208

20. Sanguineous Tumors in the Cranium .. .. .. .. .. .. .. .. .. .. .. 210

21. Iodine in Apocryphal Swellings .. .. .. .. .. .. .. .. .. .. .. .. 211

22. Tartar-Emetic in Croup .. .. .. .. .. .. .. .. .. .. .. .. .. .. 212

23. Paralysis of certain Senses .. .. .. .. .. .. .. .. .. .. .. .. .. 213

24. On the Plica Polonica .. .. .. .. .. .. .. .. .. .. .. .. .. .. .. 215

25. Annual Report of the Surgical and Ophthalmological Hospital of Berlin.. .. 218

1. A new Ligature-Instrument .. .. .. .. .. .. .. .. .. .. .. .. .. 218

2. A celebrated Styptic purchased by Godfrey and Cook .. .. .. .. .. 220

3. Fractures of the Lower jaw .. .. .. .. .. .. .. .. .. .. .. .. .. 221

4. Fractures of the Ribs .. .. .. .. .. .. .. .. .. .. .. .. .. .. 221

5. Stapheloraphy .. .. .. .. .. .. .. .. .. .. .. .. .. .. .. .. 221

6. Cæsarian operation .. .. .. .. .. .. .. .. .. .. .. .. .. .. .. 221

26. On Mucous discharges from the Uterus.. .. .. .. .. .. .. .. .. .. .. 222

1. Subacute Leucorrhœa .. .. .. .. .. .. .. .. .. .. .. .. .. .. 222

2. Chronic Leucorrhœa .. .. .. .. .. .. .. .. .. .. .. .. .. .. 223

27. Confirmation of Sir C. Bell's opinions respecting the Nerves. By Professor Muller, of Bonn.. .. .. .. .. .. .. .. .. .. .. .. .. .. .. .. 225

Letter from Sir Charles Bell to Dr. J. Johnson .. .. .. .. .. .. .. .. .. 228

28. Malformations of the Œsophagus .. .. .. .. .. .. .. .. .. .. .. .. 230

Rupture of the Œsophagus .. .. .. .. .. .. .. .. .. .. .. .. .. .. 230

### Institute of France, Academy of Sciences.

1. Mortality in Armies .. .. .. .. .. .. .. .. .. .. .. .. .. .. .. .. 231
2. Monstrosity by Diplogenesis .. .. .. .. .. .. .. .. .. .. .. .. .. .. 231
3. Lithotrity .. .. .. .. .. .. .. .. .. .. .. .. .. .. .. .. .. .. 232
4. Baregine, a Substance found in Mineral Water .. .. .. .. .. .. .. .. 232
5. On Calculous Affections .. .. .. .. .. .. .. .. .. .. .. .. .. .. 233
6. Medical Statistics .. .. .. .. .. .. .. .. .. .. .. .. .. .. .. .. 234

### Academy of Medicine.

7. Pericarditis .. .. .. .. .. .. .. .. .. .. .. .. .. .. .. .. .. .. 235

8. Amputation of Leg by Ligature .. .. .. .. .. .. .. .. .. .. .. .. 235

9. Cancer of Heart and Kidneys .. .. .. .. .. .. .. .. .. .. .. .. .. 235

10. Hermaphrodism .. .. .. .. .. .. .. .. .. .. .. .. .. .. .. .. .. 236

11. On Turning the Fœtus by the Head .. .. .. .. .. .. .. .. .. .. .. 237

12. Cholera in New Orleans .. .. .. .. .. .. .. .. .. .. .. .. .. .. 239

13. Tarentism .. .. .. .. .. .. .. .. .. .. .. .. .. .. .. .. .. .. 239

14. Extra-uterine Pregnancy .. .. .. .. .. .. .. .. .. .. .. .. .. .. 239

15. Eclampsia of young Infants .. .. .. .. .. .. .. .. .. .. .. .. .. 240

PART III.

### Clinical Review and Hospital Reports.

HOTEL DIEU.

1. Hydatid Tumours of the Wrist .. .. .. 241
2. Club-foot in New-born Children .. .. .. 242
3. Encysted Tumours on the Head .. .. .. 242
4. Syphilis and Syphiloid Diseases .. .. .. 243

HOPITAL DU MIDI.

5. Hydatid and Cancerous disease of Testicle .. .. .. 245

ST. GEORGE'S HOSPITAL.

6. Diseases of the Testis (Mr. Hawkins) .. .. .. 247

7. Fungoid disease.. .. .. .. 248

8. Removal of a Cicatrix from Burn .. .. .. 249

9. Secondary Hæmorrhage .. .. .. 250

10. Iodine in Tumor .. .. .. 251

11. Extensive Idiopathic Phlebitis .. .. .. 253

12. Wound of the Orbit by a pitch-fork .. .. .. 254

HOPITAL DE LA CHARITÉ.

13. Rheumatism .. .. .. 254
14. Chronic Icterus .. .. .. 254
15. Typhus Fever .. .. .. 254
16. Internal use of Croton Oil .. .. .. 255
17. Pericarditis after Acute Rheumatism .. .. .. 255

THE PRECEPTOR—CLINICAL LECTURES.

18. Dr. Stokes on Phthisis .. .. .. 257

19. Pleuritis Biliosa.. .. .. .. 257

20. Dysphagia .. .. .. 257

21. Diptherite .. .. .. 258

22. Laryngial Phthisis .. .. .. 258

23. Iodine in Dropsy .. .. .. 259

24. Sympathy .. .. .. 259

25. Internal Aneurism—Cases and Comments .. .. .. 261

26. Amputation in Spreading Gangrene, with Comments.. .. .. .. 265

LOCK HOSPITAL.

27. Mr. Johnson's Report on the Treatment of Gonorrhœa .. .. .. 269

### Miscellanies.

Mr. Guthrie's Case of Ligature of the Common Iliac Artery .. .. .. 278

MEDICAL REFORM.

General Observations .. .. .. 279
The Medical Gazette .. .. .. 279
Horror of Medical Reform in certain Quarters .. .. .. 280
The Scotch Doctor, Inconsistency of .. .. .. 280
One Faculty—shocking idea! .. .. .. 281
Med.-Chir. Rev. *versus* Dr. Johnson .. .. .. 282
Irish Proofs of Inconsistency ! .. .. .. 283
Westminster Medical Society .. .. .. 284

EXTRA-LIMITES.

Mr. Carmichael's Reclamation .. .. .. 285

